# Identifying metabolic limitations in the tumor microenvironment

**DOI:** 10.1126/sciadv.adq7305

**Published:** 2024-10-02

**Authors:** Guillaume Cognet, Alexander Muir

**Affiliations:** Ben May Department for Cancer Research, University of Chicago, Chicago, IL, USA.

## Abstract

Solid tumors are characterized by dysfunctional vasculature that limits perfusion and delivery of nutrients to the tumor microenvironment. Limited perfusion coupled with the high metabolic demand of growing tumors has led to the hypothesis that many tumors experience metabolic stress driven by limited availability of nutrients such as glucose, oxygen, and amino acids in the tumor. Such metabolic stress has important implications for the biology of cells in the microenvironment, affecting both disease progression and response to therapies. Recently, techniques have been developed to identify limiting nutrients and resulting metabolic stresses in solid tumors. These techniques have greatly expanded our understanding of the metabolic limitations in tumors. This review will discuss these experimental tools and the emerging picture of metabolic limitations in tumors arising from recent studies using these approaches.

## Teaser

Tools to identify metabolic stresses in tumors are improving the ability to find vulnerabilities of cancers.

## INTRODUCTION

Many solid tumors are poorly perfused because of dysfunctional tumor vasculature arising from abnormal angiogenic signaling and physical compression of blood and lymphatic vessels in the tumor (*1*–*5*). Poor perfusion in solid tumors has led to the hypothesis that the tumor microenvironment (TME) is nutrient deprived, as limited perfusion may restrict the delivery of nutrients below the metabolic requirements of both malignant and stromal cells in tumor (*6*). As a result of TME nutrient deprivation, cancer cells may rely upon metabolic adaptations to survive and grow in the TME. In contrast, cells in well-perfused normal tissues may depend less on such metabolic processes. Thus, cancer cells may have unique metabolic liabilities dictated by microenvironmental constraints that could be targeted while sparing normal cells in well-fed tissues. These metabolic liabilities are termed microenvironmental or contextual synthetic lethal targets (*7*, *8*).

The concept of therapeutically leveraging nutrient deprivation in tumors has led to much interest in identifying metabolic limitations in solid tumors (*9*), defined as those metabolic processes that are constrained by nutrient deprivation in tumors and challenge cancer cell growth and survival. However, until recently, our understanding of tumor nutrient physiology and, thus, the metabolic limitations of the TME has been limited (*10*). Advances in techniques to directly characterize nutrient conditions in the TME and methods to infer metabolic limitations in tumors have provided insight into the metabolic constraints of the TME. Here, we will discuss these newly developed tools and what they have told us about the metabolic limitations of cancers.

## EXPERIMENTAL TECHNIQUES TO IDENTIFY METABOLIC LIMITATIONS IN TUMORS

### Measuring nutrient concentrations in the TME

**Fig. 1.**
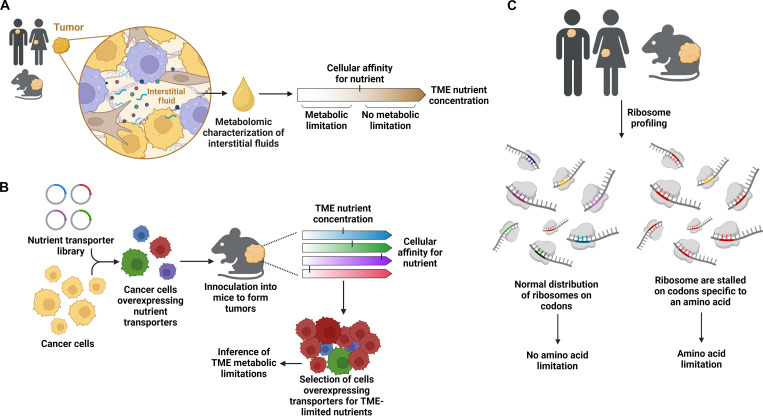
Tools to identify metabolic limitations in tumors. (**A**) Interstitial fluid can be isolated from tumors and analyzed using mass spectrometry to characterize nutrient concentrations in the tumor microenvironment. These nutrient concentrations can then be compared to known transport or cellular affinities for nutrients to infer the nutrients limiting cellular metabolism in the tumor microenvironment. (**B**) The development of CRISPR-activation libraries enables rapid screening of the transporters whose overexpression improves the fitness of cancer cells in the tumor microenvironment. Given that transporter overexpression provides a strong fitness advantage to cancer cells experiencing limitation of the cognate nutrient, such screens can identify which nutrients are limiting in the microenvironment. (**C**) The analysis of the codons engaged by ribosomes in tumors can identify which amino acids are limited and slow translation in a tumor. If an amino acid is limited in the tumor microenvironment, then ribosomes will increasingly stall at these amino acid codons. This approach has been used to identify amino acids limitations in human and animal tumors. Created with BioRender.com.

One approach for identifying metabolic limitations in tumors is to measure nutrient levels in the TME and cross-reference these levels with known cellular affinities or requirements for a given nutrient (Fig. 1A). If a nutrient in the TME is at levels below what cells require, then one could infer that this TME nutrient stress triggers metabolic limitation.

Oxygen is among the first nutrients whose TME availability has been widely quantified. The development and widespread use of tools, such as polarographic needle electrodes, to measure TME oxygen levels led to the finding that many solid tumors have regions with substantially lower oxygen availability than healthy tissues (*11*–*13*). These studies confirmed the hypothesis that cells in the TME experience deprivation of metabolic substrates and suggested lack of oxygen as one important metabolic limitation in solid tumors.

TME hypoxia has led to an interest in defining other nutrients that may be deprived in the TME. Recently, analysis of tumor interstitial fluid (TIF) has increased our understanding of nutrient availability in the TME. TIF is the local perfusate of tumors and carries nutrients to all cells in the TME (*5*). While numerous methods for isolating and analyzing TIF composition have been used to study tumor nutrient physiology for decades (*5*, *14*), early techniques had important limitations. Notably, early TIF collection methods required either surgical implantation of microperforated capsules (*15*) or use of capillaries to isolate TIF leaking from tumors after blunt dissection (*16*, *17*). Thus, these techniques were limited in application to animal models of cancer in laboratories with access to specialized devices and surgical expertise. In addition, these techniques have important experimental caveats. Surgical implantation of capsules in tumors leads to inflammatory reactions (*18*) that can affect local metabolism (*14*) and blunt dissection of tumors disrupts cells, leading to contamination of TIF with intracellular fluid (*5*). Thus, studies of TME nutrient availability could be experimentally confounded using these TIF isolation approaches. More recently, another technique, tissue centrifugation, has been developed to isolate TIF without the need for surgical device implantation or tissue dissection (*19*). Tissue centrifugation has enabled nonspecialist laboratories to isolate TIF from many animal models of cancer (*20*–*39*) and patient tumor specimens (*23*, *24*, *32*, *40*–*44*) without the experimental caveats of previous TIF isolation methods. Increased access to TIF coupled with advanced metabolomics techniques has enabled extensive quantification of the metabolites (*20*, *22*, *26*, *27*, *31*, *33*, *35*, *37*–*39*, *43*, *45*) and lipids (*24*, *25*, *27*–*29*, *34*, *45*) present in the TME. Thus, advances in TIF isolation and analysis have increased our understanding of nutrient stresses in the TME.

### Genetic approaches to identify metabolic limitations in tumors

In addition to directly measuring nutrient availability in tumors to identify TME metabolic limitations, genetic approaches can be used to infer metabolic limitations in tumors (Fig. 1B). For example, recent studies have overexpressed nutrient transporters in mouse models of cancer and identified transporters that provide a fitness advantage to cancer cells in the TME (*46*, *47*). Overexpression of a nutrient transporter provides a competitive advantage to cancer cells when deprived of the cognate nutrient (*47*). Thus, this genetic approach can be used to identify metabolic limitations in the TME that constrain cancer cell growth and fitness.

### Using cellular probes of nutrient stress to identify metabolic limitations in the TME

Identifying intracellular metabolic processes that the TME impairs can also provide insight into the metabolic limitations of tumors. Such a concept has recently been used to identify amino acid limitations in tumors. Protein translation requires a constant supply of amino acids, which are charged onto tRNAs and incorporated into elongating amino acid polymers. Protein translation stalls specifically at codons of amino acids that are limited in cancer cells (*48*–*51*). Stalling at amino acid–limited codons also reduces translation fidelity and results in ribosomal frameshifting (*52*), substitution of alternative amino acids (*50*), and premature translation termination (*53*). Thus, codon-specific reporters of ribosome stalling (*49*) or loss of translational fidelity (*51*, *52*) have been used in animal models of cancer and patient specimens to identify amino acid limitations in the TME (Fig. 1C).

## METABOLIC LIMITATIONS IN CANCERS

In this section, we will discuss the emerging portrait of metabolic limitations in the TME arising from studies using the tools outlined above. Table 1 lists the concentrations of nutrients in the TME for the nutrients discussed below as metabolically limiting in the microenvironment.

**Table 1.** Circulating and tumor microenvironmental levels in cancers. Listed are the concentrations of nutrients in the microenvironment of different cancer types that have been discussed as metabolically limiting in the tumor microenvironment (i.e., oxygen, glucose, amino acids, and vitamins). For comparison, circulating levels of each nutrient are also listed.

Nutrient (concentration unit)	Disease state	Avg. plasma concentration (range) (ref.)		Avg. tumor microenvironmental concentration (range) (ref.)	
Oxygen (mmHg)	Healthy human	100	(*54*)		
	Human brain tumor			(3–22)	(*11*)
	Human breast cancer			(2–38)	(*11*)
	Human head and neck cancer			(3–19)	(*11*)
	Human liver metastatic tumor			4	(*11*)
	Human lung cancer			(1–7)	(*11*)
	Human melanoma			6–20	(*11*)
	Human non-Hodgkin’s lymphoma			8	(*11*)
	Human pancreatic adenocarcinoma			18	(*11*)
	Human prostate cancer			(2–21)	(*11*)
	Human rectal cancer			(14–15)	(*11*)
	Human renal cell carcinoma			3	(*11*)
	Human soft tissue sarcoma			(4–22)	(*11*)
	Human uterine carcinoma			(3–17)	(*11*)
	Human vulvar cancer			(15–20)	(*11*)
Glucose (mM)	Rodent breast cancer	9.5 (7.0–10.4)	(*15*)	0.1 (0–0.3)	(*15*)
		11.7	(*16*)	4.4	(*16*)
		7.1	(*16*)	2	(*16*)
	Murine colorectal cancer	6.6 (5.5–8.6)	(*32*)	5.3 (3.0–7.8)	(*32*)
	Rodent hepatoma	11.7 (8.7–13.5)	(*15*)	0.3 (0–1.3)	(*15*)
	Murine lung cancer			3.5 (5.3–1.2)	(*20*)
				0.63 (0.54–0.74)	(*63*)
	Murine melanoma	6.2	(*29*)	0.7	(*29*)
		12.2	(*16*)	3.1	(*16*)
		9.6	(*30*)	0.7	(*30*)
	Murine pancreatic adenocarcinoma	8.2 (3–16.7)	(*20*)	3.9 (0.8–10.0)	(*20*)
		3.7 (2.9–4.9)	(*22*)	0.7 (0.2–1.2)	(*22*)
				1.96 (0.27–5.15)	(*25*)
	Human renal cell carcinoma	1.9 (0.6–4.9)	(*32*)	3.8 (1.0–8.9)	(*32*)
		5.8 (3.3–14.2)	(*43*)	1.2 (0.2–3.4)	(*43*)
				1.6 (0.8–3.2)	(*44*)
	Rodent sarcoma	11.0 (10–12.4)	(*15*)	0.4 (0–1.1)	(*15*)
		12.2	(*16*)	4.8	(*16*)
Alanine (μM)	Murine brain metastatic breast cancer	500	(*75*)	10	(*75*)
	Murine colorectal cancer	646 (542–799)	(*45*)	2871 (1950–3853)	(*45*)
	Murine lung cancer			1530 (1105–2369)	(*20*)
	Murine melanoma	366 (90–550)	(*45*)	2247 (312–3573)	(*45*)
	Murine pancreatic adenocarcinoma	847 (412–1539)	(*20*)	1023 (333–1458)	(*20*)
	Human renal cell carcinoma	447 (288–757)	(*43*)	1684 (355–4232)	(*43*)
Arginine (μM)	Murine colorectal cancer	176 (141–224)	(*45*)	118 (61–228)	(*45*)
	Murine lung cancer			9.5 (6.1–19.0)	(*20*)
	Murine lung metastasis of renal cell carcinoma			19 (1.0–68)	(*35*)
	Murine melanoma	311 (114–790)	(*45*)	413 (73–965)	(*45*)
	Murine pancreatic adenocarcinoma	130 (51–234)	(*20*)	2.3 (0.9–5)	(*20*)
	Human renal cell carcinoma	54 (21–86)	(*43*)	106 (20–248)	(*43*)
	Murine renal cell carcinoma			172 (70–352)	(*35*)
Asparagine (μM)	Murine colorectal cancer	63 (47–79)	(*45*)	255 (194–330)	(*45*)
	Murine lung cancer			126 (86–181)	(*20*)
	Murine melanoma	173 (26–628)	(*45*)	581 (89–2275)	(*45*)
	Murine pancreatic adenocarcinoma	89 (38–180)	(*20*)	108 (63–146)	(*20*)
	Human renal cell carcinoma	35 (23–46)	(*43*)	63 (16–163)	(*43*)
Aspartate (μM)	Murine brain metastatic breast cancer	20	(*75*)	4	(*75*)
	Murine colorectal cancer	20 (15–25)	(*45*)	1159 (990–1345)	(*45*)
	Murine lung cancer			281 (130–464)	(*20*)
	Murine melanoma	13.5 (6–27)	(*45*)	1422 (695–4041)	(*45*)
	Murine pancreatic adenocarcinoma	44 (14–122)	(*20*)	356 (60–519)	(*20*)
	Human renal cell carcinoma	4.3 (0.7–7.6)	(*43*)	194 (9.4–459)	(*43*)
Cystine (μM)	Murine colorectal cancer	24 (18–33)	(*45*)	41 (23–56)	(*45*)
	Murine lung cancer			39 (8–48)	(*20*)
	Murine melanoma	22 (11–43)	(*45*)	17 (9–31)	(*45*)
	Murine pancreatic adenocarcinoma	95 (30–153)	(*20*)	51 (16–111)	(*20*)
	Human renal cell carcinoma	235 (138–360)	(*43*)	317 (3.1–954)	(*43*)
Glutamate (μM)	Murine brain metastatic breast cancer	50	(*75*)	8	(*75*)
	Murine colorectal cancer	72 (55–104)	(*45*)	5164 (2885–7358)	(*45*)
	Murine lung cancer			1334 (958–1633)	(*20*)
	Murine melanoma	43 (18–90)	(*45*)	5495 (2044–19103)	(*45*)
	Murine pancreatic adenocarcinoma	81 (48–170)	(*20*)	941 (147–1300)	(*20*)
	Human renal cell carcinoma	70 (22–174)	(*43*)	1785 (55–3794)	(*43*)
Glutamine (μM)	Murine colorectal cancer	590 (550–646)	(*32*)	449 (258–827)	(*32*)
		883 (774–1046)	(*45*)	1544 (1136–1936)	(*45*)
	Murine lung cancer			709 (529–973)	(*20*)
	Murine melanoma	543 (72–846)	(*45*)	672 (74–1214)	(*45*)
	Murine pancreatic adenocarcinoma	850 (470–1379)	(*20*)	748 (233–1329)	(*20*)
	Human renal cell carcinoma			467 (163–876)	(*32*)
		498 (366–637)	(*43*)	416 (80–798)	(*43*)
Glycine (μM)	Murine brain metastatic breast cancer	300	(*75*)	14	(*75*)
	Murine colorectal cancer	401 (350–423)	(*45*)	3104 (2263–3908)	(*45*)
	Murine lung cancer			1311 (1018–2110)	(*20*)
	Murine melanoma	215 (61–354)	(*45*)	1836 (239–3815)	(*45*)
	Murine pancreatic adenocarcinoma	164 (7–354)	(*20*)	1726 (381–3654)	(*20*)
	Human renal cell carcinoma	217 (127–336)	(*43*)	757 (121–1740)	(*43*)
Histidine (μM)	Murine brain metastatic breast cancer	100	(*75*)	3	(*75*)
	Murine colorectal cancer	93 (79–107)	(*45*)	267 (200–356)	(*45*)
	Murine lung cancer			113 (91–144)	(*20*)
	Murine melanoma	76 (58–95)	(*45*)	171 (117–216)	(*45*)
	Murine pancreatic adenocarcinoma	93 (46–196)	(*20*)	89 (53–117)	(*20*)
	Human renal cell carcinoma	63 (45–84)	(*43*)	70 (22–144)	(*43*)
Isoleucine (μM)	Murine brain metastatic breast cancer	150	(*75*)	2	(*75*)
	Murine colorectal cancer	105 (94–113)	(*45*)	321 (222–442)	(*45*)
	Murine lung cancer			138 (104–169)	(*20*)
	Murine melanoma	96 (75–122)	(*45*)	170 (121–231)	(*45*)
	Murine pancreatic adenocarcinoma	155 (73–270)	(*20*)	125 (86–198)	(*20*)
	Human renal cell carcinoma	68 (22–95)	(*43*)	89 (22–230)	(*43*)
Leucine (μM)	Murine brain metastatic breast cancer	200	(*75*)	3	(*75*)
	Murine colorectal cancer	138 (125–157)	(*45*)	498 (332–650)	(*45*)
	Murine lung cancer			266 (208–326)	(*20*)
	Murine melanoma	134 (92–186)	(*45*)	288 (176–422)	(*45*)
	Murine pancreatic adenocarcinoma	298 (110–491)	(*20*)	275 (178–426)	(*20*)
	Human renal cell carcinoma	125 (76–176)	(*43*)	167 (38–418)	(*43*)
Lysine (μM)	Murine brain metastatic breast cancer	400	(*75*)	10	(*75*)
	Murine colorectal cancer	361 (259–496)	(*45*)	883 (563–1180)	(*45*)
	Murine lung cancer			363 (239–462)	(*20*)
	Murine melanoma	278 (206–385)	(*45*)	453 (239–582)	(*45*)
	Murine pancreatic adenocarcinoma	243 (98–443)	(*20*)	129 (58–214)	(*20*)
	Human renal cell carcinoma	149 (86–197)	(*43*)	203 (32–493)	(*43*)
Methionine (μM)	Murine brain metastatic breast cancer	100	(*75*)	3	(*75*)
	Murine colorectal cancer	89 (66–114)	(*45*)	242 (178–357)	(*45*)
	Murine lung cancer			138 (92–196)	(*20*)
	Murine melanoma	112 (55–215)	(*45*)	208 (89–449)	(*45*)
	Murine pancreatic adenocarcinoma	131 (43–455)	(*20*)	70 (35–107)	(*20*)
	Human renal cell carcinoma	19 (13–28)	(*43*)	24 (0.6–94)	(*43*)
Phenylalanine (μM)	Murine brain metastatic breast cancer	100	(*75*)	2	(*75*)
	Murine colorectal cancer	105 (77–130)	(*45*)	305 (209–426)	(*45*)
	Murine lung cancer			137 (109–178)	(*20*)
	Murine melanoma	67 (54–87)	(*45*)	139 (96–182)	(*45*)
	Murine pancreatic adenocarcinoma	98 (45–207)	(*20*)	76 (48–106)	(*20*)
	Human renal cell carcinoma	51 (38–62)	(*43*)	62 (18–160)	(*43*)
Proline (μM)	Murine brain metastatic breast cancer	20	(*75*)	1	(*75*)
	Murine colorectal cancer	146 (98–213)	(*45*)	808 (581–1034)	(*45*)
	Murine lung cancer			309 (228–412)	(*20*)
	Murine melanoma	86 (44–128)	(*45*)	388 (127–640)	(*45*)
	Murine pancreatic adenocarcinoma	93 (45–222)	(*20*)	114 (52–164)	(*20*)
	Human renal cell carcinoma	145 (83–247)	(*43*)	137 (32–279)	(*43*)
Serine (μM)	Murine brain metastatic breast cancer	200	(*75*)	4	(*75*)
	Murine colorectal cancer	192 (137–217)	(*45*)	728 (496–965)	(*45*)
	Murine lung cancer			252 (154–384)	(*20*)
	Murine melanoma	143 (88–217)	(*45*)	443 (241–637)	(*45*)
	Murine pancreatic adenocarcinoma	37 (0.5–133)	(*20*)	191 (81–265)	(*20*)
	Human renal cell carcinoma	90 (60–133)	(*43*)	168 (31–508)	(*43*)
Threonine (μM)	Murine brain metastatic breast cancer	200	(*75*)	5	(*75*)
	Murine colorectal cancer	224 (192–275)	(*45*)	717 (447–965)	(*45*)
	Murine lung cancer			465 (313–823)	(*20*)
	Murine melanoma	180 (131–234)	(*45*)	480 (328–627)	(*45*)
	Murine pancreatic adenocarcinoma	316 (122–919)	(*20*)	235 (120–314)	(*20*)
	Human renal cell carcinoma	94 (41–139)	(*43*)	138 (27–348)	(*43*)
Tryptophan (μM)	Murine colorectal cancer	103 (87–116)	(*45*)	190 (124–279)	(*45*)
	Murine lung cancer			75 (50–99)	(*20*)
	Murine melanoma	125 (84–201)	(*45*)	81 (0–170)	(*45*)
	Murine pancreatic adenocarcinoma	82 (28–151)	(*20*)	28 (9.7–71)	(*20*)
	Human renal cell carcinoma	40 (20–67)	(*43*)	19 (0.8–59)	(*43*)
Tyrosine (μM)	Murine brain metastatic breast cancer	200	(*75*)	4	(*75*)
	Murine colorectal cancer	114 (90–140)	(*45*)	321 (208–429)	(*45*)
	Murine lung cancer			126 (86–179)	(*20*)
	Murine melanoma	69 (46–107)	(*45*)	121 (75–168)	(*45*)
	Murine pancreatic adenocarcinoma	93 (33–225)	(*20*)	55 (25–110)	(*20*)
	Human renal cell carcinoma	71 (42–98)	(*43*)	78 (18–192)	(*43*)
Valine (μM)	Murine brain metastatic breast cancer	400	(*75*)	40	(*75*)
	Murine colorectal cancer	199 (172–239)	(*45*)	600 (369–814)	(*45*)
	Murine lung cancer			329 (255–409)	(*20*)
	Murine melanoma	166 (78–221)	(*45*)	339 (162–516)	(*45*)
	Murine pancreatic adenocarcinoma	201 (78–360)	(*20*)	142 (82–222)	(*20*)
	Human renal cell carcinoma	204 (103–308)	(*43*)	162 (33–369)	(*43*)
Pyridoxine (ng/ml)	Murine pancreatic adenocarcinoma	0.27	(*36*)	0.14	(*36*)
Pyridoxal phosphate (ng/ml)	Murine pancreatic adenocarcinoma	0.45	(*36*)	0.29	(*36*)

### Oxygen

As discussed above, hypoxia is one of the most widely documented TME nutrient stresses. The partial pressure of oxygen levels in many tumor types is ~10 mmHg. Some particularly hypoxic tumors, such as pancreatic cancers, have oxygen tensions as low as 2 mmHg (*11*). This is substantially less than most healthy tissues, which have oxygen tensions of ~30 to 50 mmHg (*11*).

However, before concluding that hypoxia causes metabolic limitations in cancers, it is necessary to compare the TME level of oxygen with known cellular affinities and requirements for oxygen. Many oxygen-using metabolic processes, such as mitochondrial respiration, have high affinities for oxygen (*54*). Thus, the TME of even extremely hypoxic tumors is oxygenated above the requirements of the respiratory chain and does not prevent cellular respiration (*55*, *56*). However, many metabolic enzymes have affinities for oxygen that could lead to substrate limitation, and TME hypoxia has been shown to impair their activity (*54*). Furthermore, prolonged TME hypoxia triggers regulatory pathways such as the hypoxia-inducible factor pathway that constrain metabolic pathway activity (*57*, *58*). Thus, hypoxia can trigger metabolic limitations in the TME by constraining the activity of certain metabolic pathways. In particular, hypoxia has been found to metabolically limit cancer cells by impairing aspartate synthesis (*46*, *59*), inhibiting unsaturated fatty acid synthesis (*60*), and triggering the degradation of heme (*61*, *62*). However, more work will need to be done to define the complete set of metabolic processes limited by TME hypoxia and whether these limitations are driven by substrate limitation or hypoxic signaling.

### Glucose

TIF analysis indicates that glucose is depleted in the TME of many tumor types (*15*, *16*, *20*, *22*, *29*, *30*, *63*). Furthermore, transporter overexpression screens indicate that glucose may be limiting in the TME of some animal cancer models (*47*). Thus, glucose deprivation is a common feature of many tumors. However, glucose is not depleted in the TME of all tumor types (*32*, *43*, *44*), and overexpression of glucose transporters does not potentiate cancer cell growth in every animal model of cancer (*47*). In addition, TME glucose levels change markedly over the course of disease progression (*29*). Thus, while glucose deprivation may be common in cancers, it is not a universal feature of every tumor type and stage.

As discussed above with TME hypoxia, it is also necessary to consider cellular affinities and requirements for glucose to determine whether glucose deprivation constitutes a metabolic limitation of the TME. The most severe TME depletion of glucose has been observed in animal models of pancreatic cancer and melanoma with glucose concentrations of around 0.6 mM (*22*, *29*, *30*). However, glucose transporters have low millimolar affinities for glucose (*64*), and glucose concentrations of as low as 0.5 mM are sufficient to support nearly maximal cellular proliferation (*65*). Thus, while glucose can be depleted in the TME, it may not be depleted enough to trigger metabolic limitation at the substrate level. However, glucose deprivation activates numerous signaling pathways that regulate cellular metabolism, such as the mechanistic target of rapamycin complex 1 pathway, adenosine 5′-monophosphate–activated protein kinase (AMPK) signaling, and the integrated stress response (*66*, *67*). Activation of such pathways upon glucose starvation regulates nutrient import, macromolecule synthesis, energy metabolism homeostasis, and other metabolic processes. Thus, glucose deprivation may cause metabolic limitations by regulating cellular signaling pathways that affect metabolism. In one example, AMPK activation upon glucose starvation of cancer cells leads to phosphorylation and inactivation of acetyl–coenzyme A carboxylase (ACC), a key enzyme in lipid synthesis (*68*). AMPK inhibition of ACC suppresses lipid synthesis and renders cancer cells lipid auxotrophic (*68*). Thus, glucose regulation of AMPK can trigger a lipid metabolic limitation in cancer cells. Further studies will be required to understand how pathophysiological glucose levels in the TME limit cancer cell metabolism via these signaling interactions.

### Amino acids

#### Arginine

Arginine is depleted in the TME of pancreatic cancers (*21*, *31*, *69*) and lung metastases of kidney tumors (*35*). TIF arginine levels in these tumors are approximately an order of magnitude lower than the cellular affinity for arginine (*70*, *71*) and below concentrations required for cellular proliferation (*47*). In both cases, cancers require adaptations to cope with this limitation. For example, pancreatic and kidney cancer cells adapt by increasing the synthesis of arginine (*21*, *35*), and pancreatic cancers also use alternative metabolic routes to generate arginine-derived metabolites like polyamines (*31*). Infiltrating lymphocytes cannot adapt to TME arginine deprivation (*72*), and arginine metabolic limitation is a major barrier to their function (*69*). Thus, arginine deprivation is a metabolic limitation in some tumor types.

#### Serine

Nutrient levels in the cerebrospinal fluid and the brain interstitium are tightly regulated by the choroid plexus (*73*) and the blood-brain barrier (*74*). This leads to restricted levels of many nutrients in the brain, including the amino acid serine (*75*). Serine is at levels in the brain well below the requirements of many cancer cells for growth (*47*, *75*, *76*). Tumors metastasizing to the brain require increased serine synthesis to overcome this limitation (*75*). Thus, serine deprivation is a metabolic limitation for tumors localized in the brain.

#### Tryptophan

Tryptophan is readily degraded by dioxygenases such as indoleamine 2,3-dioxygenase and tryptophan 2,3-dioxygenase whose expression is induced by inflammatory signaling in some tumors (*77*, *78*). Thus, tryptophan can be depleted in some tumors. Tryptophan depletion impairs protein translation in cancer cells (*50*) and results in misincorporation of phenylalanine into tryptophan tRNAs and proteins (*51*). Consistent with tryptophan depletion being a metabolic limitation in inflamed TMEs, tryptophan-phenylalanine misincorporation occurs specifically in tumors with high levels of inflammation (*51*). Thus, tryptophan deprivation in inflamed tumors metabolically limits protein translation and translation fidelity. How tryptophan depletion metabolically limits cancer cells aside from impairing protein translation remains to be determined.

#### Proline

Analysis of ribosome stalling and tRNA charging in patient renal cell carcinomas and animal models of breast cancer indicates that proline levels are limiting for translation in these tumors (*49*). In addition to endogenous proline limitation, dietary restriction of proline can trigger proline and translation limitation in other tumor types as well (*79*). Tumors adapt by increasing the synthesis of proline, which can be targeted to slow tumor progression (*49*). Thus, in certain tumor types, proline availability may be a metabolic limitation to which cancer cells must adapt.

#### Glutamine

Glutamine restriction in cancer cells leads to translational errors, including ribosomal frameshifting (*52*). Genetically encoded ribosomal frameshifting reporters have been developed to identify cells experiencing glutamine deprivation–induced protein translation errors (*52*). The use of these reporters in animal models of pancreatic cancer indicates that small regions of tumors are metabolically limited by glutamine deprivation at certain stages of tumor development (*52*).

### Lipids

Lipids are normally available at high levels in healthy tissues and tumors. However, as discussed above in the case of serine, brain interstitial fluid and cerebrospinal fluid contain restricted levels of many nutrients, including lipids (*34*). Thus, lipid depletion is a metabolic limitation for tumors growing in the brain. Cancers growing in this compartment require adaptations to increase de novo synthesis of lipids to overcome this metabolic limitation (*34*, *80*).

### Vitamins and micronutrients

Most analyses of nutrient stresses in TMEs have focused on high-demand nutrients such as glucose, amino acids, and lipids that are large contributors to cellular biomass (*81*). However, recent studies also indicate that micronutrients may also be deprived in the TME and drive important metabolic limitations in cancers.

For example, vitamin B6 levels are strongly depleted in the TIF of pancreatic cancers to levels insufficient for natural killer (NK) cell function (*36*). Furthermore, dietary vitamin B6 supplementation improves NK cell function in the pancreatic ductal adenocarcinoma TME, demonstrating that vitamin B6 deprivation is a TME metabolic limitation for certain classes of immune cells.

In another example, iron availability is substantially lower in cerebrospinal fluid than in the circulation. Iron at cerebrospinal fluid levels is limiting for cancer cell growth (*82*). Leptomeningeal metastases adapt to this limitation by increasing the activity of iron-scavenging pathways, which are required for metastatic progression (*82*). Thus, iron deprivation is a metabolic limitation for metastatic spread to the leptomeninges. These studies highlight that micronutrient availability can be a metabolic limitation of the TME. Further analysis of TME micronutrients is warranted to understand how the availability of these nutrients drives metabolic limitations in cancers.

## FUTURE DIRECTIONS

Recent technological developments have substantially improved the ability to identify metabolic limitations of cancers. The newly gained ability to find metabolic limitations will be critical to identify the adaptations that tumors engage to fuel growth and progression despite TME metabolic constraints. These adaptations are ideal TME or contextual synthetic lethal targets and could improve upon existing metabolic cancer drugs that often have limited therapeutic windows given the requirements of many existing metabolic targets for tissue function (*83*, *84*). Thus, identifying TME metabolic limitations could prove critical for therapeutically leveraging metabolism for cancer therapy. What will be necessary to translate our understanding of TME metabolic limitations into efficacious therapeutic targets?

First, the emerging picture of the TME suggests that metabolic limitations are heterogeneous. Not all metabolic limitations are shared by all tumor types, and the limitations can change during disease progression. To realize the potential of identifying efficacious metabolic targets driven by TME metabolic limitations, it will be necessary to use the discussed tools and methods across tumor types and stages to determine which metabolic limitations and adaptations are relevant to a given tumor type and stage. In addition, most techniques described in this review characterize the TME nutrient landscape at the whole tumor level. Further development and use of methods that provide spatial resolution of the nutrient landscape within tumors, such as mass spectrometry imaging (*85*), will be necessary to characterize the extent to which a metabolic limitation occurs in a tumor.

Second, in this review, we have only considered metabolic limitations driven by nutrient deprivation in the TME. However, many metabolites accumulate in the TME and can have diverse effects on tumor metabolism. In some cases, these accumulating metabolites can serve as alternative substrates that buffer metabolic limitations driven by the deprivation of other nutrients (*22*, *46*). For instance, high levels of uridine in the pancreatic TME can be salvaged by cancer cells to alleviate the metabolic limitations caused by glucose depletion in these tumors (*22*). In other cases, metabolites accumulating in the TME can act as inhibitors of different metabolic processes and drive metabolic limitations (*86*, *87*). Thus, tools must be developed to determine how metabolites that accumulate in the TME impinge upon cellular metabolism and drive or alleviate metabolic limitation in the TME. These further advancements will identify metabolic limitations that can be exploited for cancer therapies and the contexts in which these therapies will be efficacious.
